# Latent Autoimmune Diabetes in Adults: A Diagnostic Challenge in Elderly Patients

**DOI:** 10.7759/cureus.84457

**Published:** 2025-05-20

**Authors:** Filipa Monteiro, Catarina Artilheiro, Tetiana Shara, Maria Teresa Andrade, Leticia Santos, Andreia Nunes, Sara Ramalho, Conceição Escarigo

**Affiliations:** 1 Internal Medicine, Garcia de Orta Hospital, Almada, PRT

**Keywords:** anti-gad antibody, auto-imune diseases, endocrinology and diabetes, insulin-resistance, latent autoimmune diabetes in adults

## Abstract

Diabetes mellitus (DM) is a chronic condition with several subtypes that differ in etiology and clinical progression. Type 2 diabetes (T2DM) is the most commonly diagnosed form and is closely linked to obesity and physical inactivity, while type 1 diabetes (T1DM) is largely autoimmune in origin. A less understood subtype, latent autoimmune diabetes in adults (LADA), shares characteristics of both T2DM and T1DM and is often misdiagnosed as T2DM. This article presents three clinical cases of elderly patients misdiagnosed with T2DM, but who actually had LADA, highlighting the challenges associated with its early identification and the importance of more accurate diagnostic approaches.

## Introduction

Diabetes mellitus (DM) is a chronic metabolic disease condition, affecting millions globally, characterized by hyperglycemia resulting from defects in insulin secretion, insulin resistance, or both. While type 2 diabetes (T2DM) constitutes over 90% of DM cases, there are patients where the condition does not fit the classic characteristics of either T2DM or type 1 diabetes (T1DM), leading to misdiagnoses. Understanding the disease's heterogeneous nature is critical, particularly in cases like latent autoimmune diabetes in adults (LADA). LADA is a common but understudied diabetes subtype, often evolving with progressive metabolic deterioration and inadequate glycemic control. It is an autoimmune form of diabetes, sometimes called type 1.5 diabetes, since it is a heterogeneous entity that combines features of both T2DM and T1DM, and is often misdiagnosed as T2DM due to its similar clinical presentation [1,2].

T1DM and LADA must be viewed as a spectrum of immune-mediated pancreatic destruction and insufficiency, representing autoimmune diabetes [3]. While β-cell dysfunction is less severe at onset compared to T1DM, it advances more rapidly than in T2DM and it is yet unclear which factors are associated with progression [4]. Unlike T1DM, which presents frequently in childhood, LADA is usually diagnosed in adulthood with a slower autoimmune-mediated β-cell destruction.

LADA is particularly challenging to diagnose in elderly patients because it typically presents after age 30 and may initially not show classic signs of autoimmunity, such as high levels of antibodies. The diagnosis of LADA requires a differentiated approach, considering the presence of autoantibodies, the patient’s clinical history, decline in C-peptide levels and their response to treatment [1].

## Case presentation

Case 1

This case presents a 74-year-old woman with a maternal family history of T2DM and a known diagnosis of T2DM since she was 59 years old. At the time of diagnosis, her glycated hemoglobin (HbA1c) was 11% and she was medicated with metformin 850 mg + sitagliptin 50 mg twice daily. Due to the maintenance of off-target blood glucose levels, it was decided to start insulin Levemir 8 UI, which was discontinued two years later due to the improvement in HbA1c to 7.9% and gliclazide 60 mg daily was initiated. The patient also had a medical history of dyslipidemia and rheumatoid arthritis and was medicated with prednisolone 2.5 mg, methotrexate, and atorvastatin 20 mg. Despite the therapy instituted, the patient’s metabolic condition deteriorated again and developed diabetic neuropathy. Basal insulin therapy with glargine 8 UI was introduced by her attending physician, but her glycemic profile deteriorated further, accompanied by weight loss. The patient was referred to an internal medicine clinic consultation for investigation of weight loss (five kilograms in three months) and weighed 44 kgs (18.8 kg/m² of body mass index (BMI)). An endoscopy, colonoscopy, and abdominal-pelvic computed tomography (CT) scan were performed, and no abnormalities were found. Her HbA1c was 9.5%, fasting glucose levels ranged from 120 to 160 mg/dL, and postprandial levels between 250 and 380 mg/dL. Given her disease evolution, with weight loss, catabolic symptoms (polyphagia, polydipsia, and polyuria), along with a history of years of poorly controlled metabolic management, LADA was considered. Oral antidiabetic agents were discontinued, and a basal-bolus insulin regimen was initiated. Laboratory findings revealed a low C-peptide level of 0.27 ng/mL and positive anti-glutamic acid decarboxylase antibodies (GADA) of 18.1 U/mL (Table 1). The presence of positive GADA confirmed the diagnosis of LADA. Her basal-bolus insulin therapy was optimized, and at follow-up consultation three months later, her HbA1c had decreased to 6.3%, without major hypoglycemia. She had also regained weight, demonstrating effective management and improved glycemic stability.

Case 2

A 69-year-old woman presented with a medical history of obesity, hypertension, dyslipidemia, ischemic heart disease, and a 15-year history of T2DM. Despite being treated with metformin 1000 mg twice daily and insulin glargine 16 UI daily, she experienced poor metabolic control. The patient was admitted to the emergency department with a history of epigastric pain and vomiting for 12 hours. On physical examination, she was conscious, tachypneic with a Kussmaul breathing pattern and had tenderness on palpation of the upper abdominal quadrants. A blood gas analysis was performed, which showed metabolic acidosis with a pH of 6.89, blood glucose of 389 mg/dL, and lactate of 3.4 mmol/L. She had a ketonemia of 4.5 mmol/L. A diagnosis of diabetic ketoacidosis was made, and appropriate treatment, such as intravenous fluids and insulin, was initiated. On the fifth day of hospitalization, her HbA1c was 10.1%, C-peptide was <0.1 ng/mL, and blood glucose was 379 mg/dL. Upon discharge, metformin 1000 mg twice daily and premixed insulin 20 UI at breakfast, 10 UI at lunch, and 10 UI at dinner were prescribed, and she was re-evaluated two months later in a follow-up appointment. The HbA1c was 11.3%, and the autoimmune study was positive for GADA (78.9 U/mL, which is presented in Table 1), confirming the diagnosis of LADA. Given the metabolic deterioration, the previous insulin regimen was discontinued and a basal-bolus insulin regimen was started with 40 UI of insulin degludec daily and insulin aspart at mealtimes according to the provided schedule. After three months, the patient had an HbA1c of 7.8% with no evidence of microvascular complications.

Case 3

We present an 81-year-old woman with a known history of diabetes for five years, hypertension and osteoporosis. Upon the diagnosis of diabetes, she started oral antidiabetic therapy introduced sequentially (metformin, dapagliflozin, and gliclazide), achieving good metabolic control with this treatment (HbA1c 6.8%). However, she later developed complaints of weight loss (approximately 10 kgs in one year, initial weight 48 kgs) and underwent an investigation with her attending physician, including abdominal and pelvic computed tomography (CT) scans, endoscopy, and colonoscopy, all of which showed no abnormalities. She presented to the emergency department with a history of approximately one month of polydipsia, polyuria, and polyphagia, associated with poor glycemic control, with fasting capillary glucose values of around 270 mg/dL and postprandial glucose of 420 mg/dL. Blood tests showed glucose of 380 mg/dL, with mild ketonemia (β-hydroxybutyrate 0.2 mmol/L) but no metabolic acidosis or other significant changes, including ionic or renal function abnormalities. Upon discharge, she continued metformin 1000 mg + dapagliflozin 5 mg twice daily, started insulin glargine 8 UI per day and was referred to a diabetes clinic. At this appointment, the patient had an HbA1c of 7.1%, with occasional capillary blood glucose readings of 248 mg/dL. Given the disease progression, LADA was suspected and investigations revealed a low C-peptide level (0.20 ng/mL), glucose (106 mg/dL), positive GADA (89.7 U/mL), and zinc transporter protein antibodies (>2000 U/mL) (Table 1). After the diagnosis of LADA, basal insulin therapy was continued without implementing a basal-bolus regimen due to the patient's limited self-management capacity and frailty.

**Table 1:** Laboratory findings at diagnosis. HbA1c: glycated hemoglobin; GADA: glutamic acid decarboxylase antibodies.

Parameter tested	Reference ranges	Case 1	Case 2	Case 3
HbA1c	4.5-6.5%	9.5	11.3	7.1
C-peptide	0.37-1.47 nmol/L	0.27	<0.10	0.20
GADA	<10.0 U/mL	18.1	78.9	89.7
Zinc transporter protein antibodies	<15 U/mL	-	-	>2000

## Discussion

LADA typically presents with an adult onset (>30 years) and symptoms of hyperglycemia. It can vary from diabetic ketoacidosis to mild non-insulin-requiring diabetes, as seen in case reports, which frequently detail these presentations [5]. Patients often exhibit less pronounced metabolic syndrome and lower BMI compared to those with T2DM [1,6]. Many cases involve co-existing autoimmune conditions, such as autoimmune polyendocrine syndrome, celiac disease, abnormal thyroid function, and Graves’ disease [5,7].

LADA often progresses from an initial insulin-independent phase to insulin dependence within approximately five years, and autoantibodies against pancreatic beta cells are well characterized [6]. At the time of diagnosis, insulin secretion capacity is often similar in patients with LADA and T2DM. LADA patients may have enough β-cell function to be controlled only with diet and oral antidiabetic therapy at onset [8]. Evidence suggests a need for early intervention with therapies aimed at preserving β-cell function, as delayed treatment exacerbates autoimmune destruction and increases the risk of complications [1].

The three LADA case reports illustrate the diagnostic and management challenges posed by this atypical form of diabetes and how misclassification is significant [2,9,10]. Despite variations, patients typically present low BMIs, progressive metabolic deterioration with hyperglycemia symptoms, and inadequate response to oral antidiabetics [2,7]. Positive GADA and low C-peptide levels were pivotal in confirming LADA diagnoses across cases, highlighting that autoimmune and beta-cell function assessments may be cost-effective tools in targeted patients with adult-onset diabetes [1,10].

Management strategies are centered on insulin therapy, with basal-bolus regimens tailored to individual capacities and clinical needs. Remarkable improvements in glycemic control underscore the effectiveness of early insulin intervention. The cases emphasize the necessity of early appropriate screening of LADA to avoid misclassification, including of patients who do not fit the typical T2D profile or who present unexpected clinical courses such as poor glycemic control or becoming insulin-dependent sooner than expected [10]. The best therapeutic approach must be tailored to each patient's profile, implementing the lowest insulin doses to keep metabolic control and adapting when insulin-secretory capacity of the β-cell is lost [10].

## Conclusions

The three cases presented emphasize the importance of early diagnosis of LADA in elderly patients, a condition that, while typically diagnosed in younger adults, is rarely considered in older individuals. These cases underline the need for a differentiated approach and heightened clinical suspicion of LADA in older patients, particularly when there is a history of poor glycemic control, limited response to oral therapies, or rapid progression to insulin dependency. Recognizing that LADA can affect older adults is crucial to avoid misdiagnosis, inappropriate treatment, and poor quality of life. The confirmation of the diagnosis through positive antibodies and low C-peptide levels is essential for directing appropriate treatment. Early initiation of insulin-based therapies demonstrates significant benefits, showing that early intervention can improve glycemic control and prevent severe complications even in older age.
